# Hemorrhagic Necrotizing Cholecystitis With Cholecystocolonic Fistula: A Case Report

**DOI:** 10.7759/cureus.32187

**Published:** 2022-12-04

**Authors:** Awadh AlQahtani, Retaj Alkhawaja, Wadha S AlOtaibi, Shahad N Alanazi, Yazeed AlKhayyal, Bushr Mrad, Maha-Hamadien Abdulla, Noura AlHassan, Thamer Bin Traiki

**Affiliations:** 1 Department of Surgery, College of Medicine, King Saud University, Riyadh, SAU; 2 Colorectal Research Chair, Department of Surgery, College of Medicine, King Saud University, Riyadh, SAU

**Keywords:** cholelithiasis, hemorrhagic cholecystitis, cholecystocolonic fistulas, cholecystoenteric fistulas, acute cholecystitis

## Abstract

Cholecystocolonic fistula (CCF) and hemorrhagic cholecystitis are rare complications of gallstones that have a wide range of non-specific symptoms and clinical severity. We present a case of a 74-year-old woman on warfarin who presented to the emergency department with a 10-day history of abdominal pain, vomiting, and watery diarrhea. Her abdomen was distended with generalized tenderness and palpable mass in the right lower quadrant. Laboratory tests revealed leukocytosis and an elevated international normalized ratio (INR). After admission and imaging, exploratory laparotomy showed hemorrhagic cholecystitis with CCF in the cecum. There was no pus or stool contamination. A cholecystectomy followed by right hemicolectomy with primary ileocolic anastomosis was performed. The postoperative course was uneventful, and the patient was discharged in stable condition.

The presence of hemorrhagic cholecystitis in conjunction with CCF could lead to significant consequences such as hemorrhagic and septic shock in older patients with comorbidities. It is crucial to identify and intervene early before clinical deterioration.

## Introduction

The prevalence of gallstones among adults in developed countries is 10-15%. Many risk factors were reported in the literature to be associated with gallstone disease. Risk factors include but are not limited to family history, genetic predisposition, ethnic background, female sex, patient age, obesity, rapid weight loss, metabolic syndrome, medications, and lifestyle changes [1,2]. Most gallstones remain silent, whereby up to 25% of cases become symptomatic in the form of cholecystitis, cholangitis, or biliary pancreatitis [1].

Cholecystoenteric fistulas are a rare consequence of gallstone disease. According to a current case series involving more than 10,000 patients who underwent cholecystectomy, cholecystocolonic fistulas (CCFs) were found in 0.06-0.14% of cases [3]. CCF is considered the second most common type of cholecystoenteric fistula after cholecystoduodenal fistulas [3]. Costi et al. reviewed articles published between 1950 and 2006 for the possible pathogenic mechanisms of CCF. Longstanding inflammation caused by gallstones was found to be the most common mechanism [3,4], although factors like gastric surgery, cholecystostomy, malignancy, and trauma have also shown an association with fistula formation [3,5]. Also, sometimes cholecystoduodenal fistula leads to gall stone ileus.

From a pathological perspective, CCF secondary to gallstones can be explained by two theories. The first theory is that acute calculus cholecystitis leads to inflammation and adhesion of the gallbladder wall to the serosal surface of the adjacent structures, resulting in ischemia that eventually becomes gangrenous. Subsequently, the gallbladder contents penetrate the gangrenous wall due to the increased pressure on the gallbladder [4,6]. The other theory explains CCF by pressure necrosis caused by a gallstone impaction [6]. Patients usually present with nonspecific symptoms, where diarrhea is the most prominent presentation and is related to the excessive presence of bile acid in the colon [3,7]. Moreover, malabsorption of fat-soluble vitamins is caused by a lack of bile acids and impaired fat emulsification. Vitamin K malabsorption can lead to high bleeding tendency and elevated international normalized ratio (INR) in CCF [7,8]. In rare cases, CCF can cause recurrent unexplained hematochezia associated with gallbladder bleeding [9,10].

Here, we report the case of an elderly female patient with multiple medical comorbidities presenting with abdominal pain, diarrhea, and a high INR. These are two uncommon pathologies, hemorrhagic cholecystitis and CCF in the cecum. 

## Case presentation

A 74-year-old woman with a history of mitral valve replacement (MVR) 13 years prior with chronic treatment of 6 mg warfarin presented to the emergency department with a 10-day history of right upper quadrant abdominal pain, persistent vomiting, and watery diarrhea.

On physical examination, the patient was afebrile and vitally stable. The patient was tachycardic, initially reaching 120 bpm, and then improved to 80-90 bpm with analgesia. The abdomen was distended with generalized tenderness, mainly on the right side with a negative Murphy’s sign. A mass in the right lower quadrant was palpated. Her work-up revealed a raised white blood cell count of 13,700 cells/μL, hemoglobin level of 9.8 g/dL, and an INR of 16.00.

**Figure 1:**
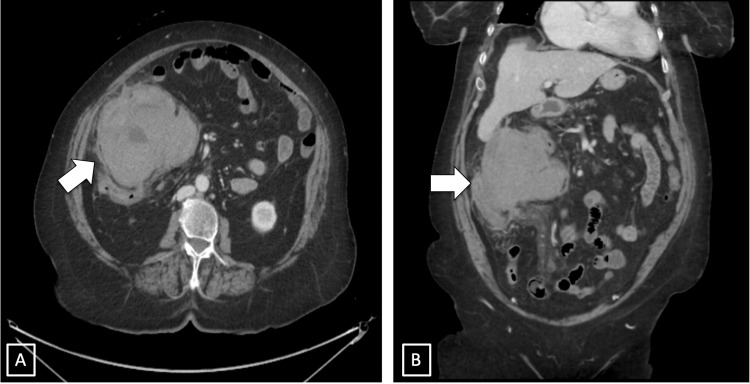
CT abdomen (A) axial view showing a heterogenous mass (large arrow) inseparable from the right colon. (B) Coronal view heterogenous mass (large arrow) away from liver bed.

The patient was admitted, and nothing by mouth (NPO) was maintained with intravenous fluid and analgesia. Cardiology was consulted because of the high INR. A computed tomography (CT) scan with intravenous contrast of the abdomen and pelvis revealed a heterogeneously enhancing mass in the right lower quadrant indivisible from the small and large bowel, with the cystic duct tethered toward the mass (Figure 1).

Additionally, there were regional peritoneal deposits, lymphadenopathy, and a small volume of hemoperitoneum, which could be related to the described mass.

As malignancy was suspected, the surgical oncology team advised a chest CT and colonoscopy for proper staging, which showed cardiomegaly and a small volume of right-sided pleural effusion with no evidence of intrathoracic metastases. The gastroenterology team considered colonoscopy unnecessary at this stage. Abdominal magnetic resonance imaging (MRI) showed a large hemorrhagic lesion on the right side of the abdomen surrounding a thickened gallbladder abutting the right side of the colon.

**Figure 2:**
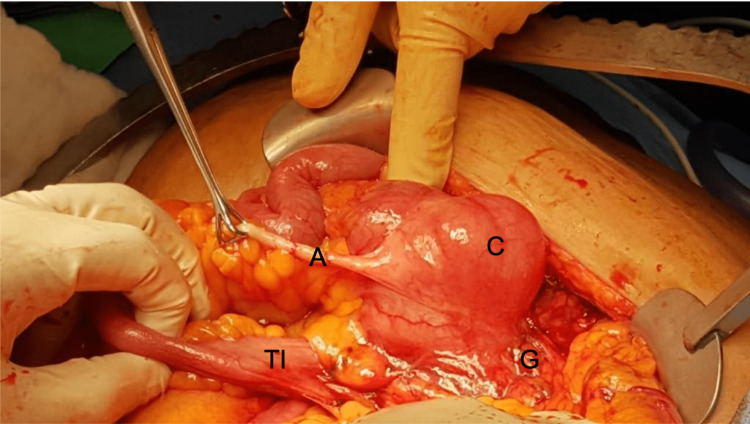
Gallbladder adhered to the cecum. C: Cecum, G: Gallbladder, TI: Terminal ileum, A: Appendix

**Figure 3:**
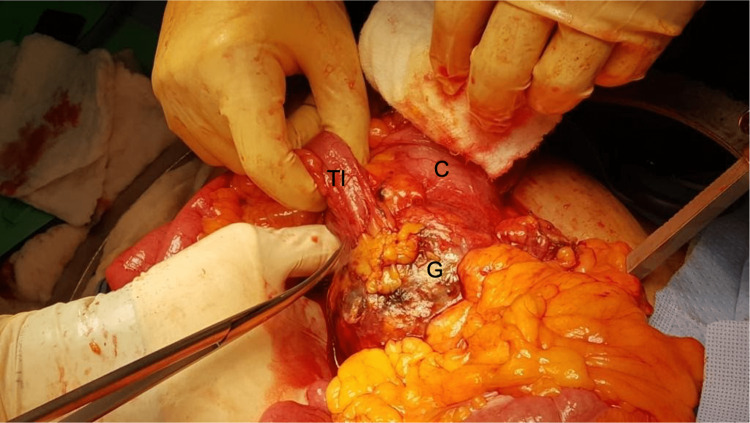
Cholecystocolonic fistula. C: Cecum, G: Gallbladder, TI: Terminal ileum

Exploratory laparotomy was performed, due to the lack of improvement, through an upper midline incision. The decision to proceed to exploratory laparotomy was due to the patient fragility as she could not tolerate a long laparoscopic procedure and moreover, there was a possibility of colon or gallbladder malignancy. The gallbladder was separated from the liver, inferiorly migrated and adhered to the cecum (Figures 2, 3).

**Figure 4:**
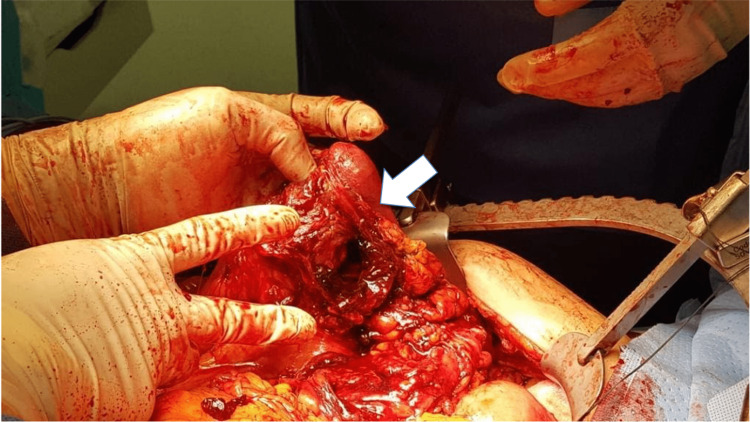
Fistula opening at the colon (arrow) after cholecystectomy.

The cystic duct stump was auto avulsed from the gallbladder and controlled using a silk stitch. The colon was dissected from the gallbladder and a fistula opening was identified (Figure 4).

**Figure 5:**
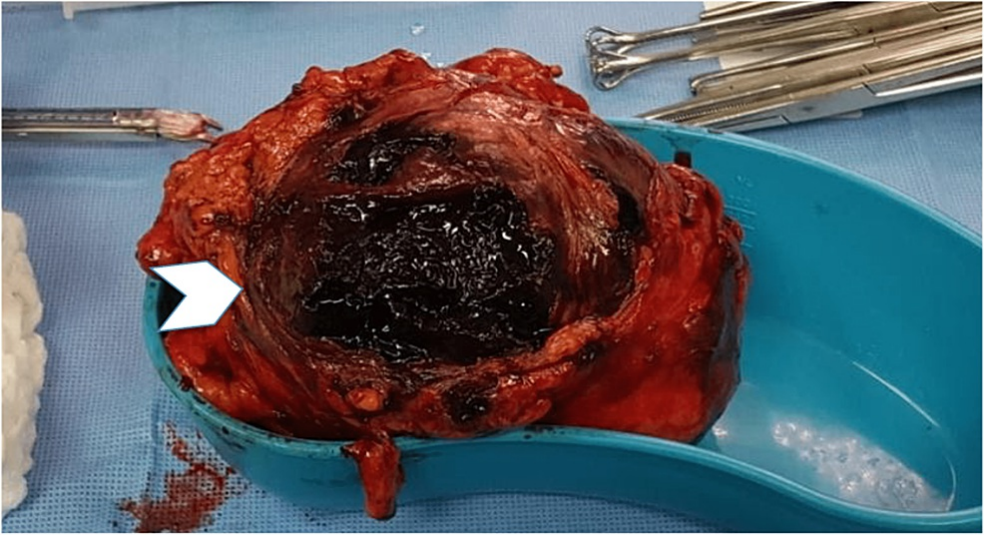
Gallbladder specimen, fistula opening with intra-luminal blood clots (arrowhead)

The gallbladder was inflamed, with a thickened wall and blood clots within the lumen (Figure 5).

We elected to do oncological resection as there was a possibility of hidden colon malignancy. Cholecystectomy followed by right hemicolectomy was performed with primary side-to-side ileocolic anastomosis.

During the postoperative course, the patient was transferred to the intensive care unit (ICU) for two days and then transferred to the surgical ward. The patient was discharged in a stable condition. One month later, she visited the outpatient clinic with no complaints and a well-tolerated diet.

The surgical pathology report revealed the right colon to have focal ulceration, fistula tract formation, granulation tissue, submucosal edema, congestion, and serosal surface exudates. These findings were observed mainly in the cecum and pericecal regions, and no evidence of malignancy was found. The gallbladder showed acute hemorrhagic necrotizing cholecystitis with a large blood clot occluding and distending the gallbladder lumen. A fistulous tract with granulation tissue and fibrosis was also identified.

## Discussion

Both CCF and hemorrhagic cholecystitis are rare gallstone complications that can lead to considerable symptoms and have a major impact on patient health [8-10]. Early intervention is crucial to avoid life-threatening consequences [11-13]. In this report, we present the case of an elderly woman with multiple comorbidities who underwent exploratory laparotomy, cholecystectomy, and right hemicolectomy for CCF and hemorrhagic cholecystitis. 

Hemorrhagic cholecystitis clinically manifests with right upper quadrant pain, positive Murphy’s sign, impaired liver function, and leukocytosis [14,15]. Haemobilia, jaundice, and melena have also been documented as uncommon presentations [15,16]. Transmural inflammation causes mural necrosis and ulceration, followed by hemorrhage into the gallbladder lumen. Clots can develop that contribute to distension and subsequent gallbladder perforation in the abdomen [14]. The hemorrhage originates from either the gallbladder itself or the liver in cases of transhepatic perforation [17]. A literature review of articles from 1987 to 2017 by Tarazi et al. identified that the majority of hemorrhagic cholecystitis cases were associated with other medical comorbidities and the use of anticoagulants or antiplatelet medications [15]. Other risk factors included uremia, chronic kidney disease, atrial fibrillation, and chronic obstructive pulmonary disease in patients using steroids chronically [15].

Imaging techniques play a crucial role in the early diagnosis of hemorrhagic cholecystitis. Ultrasonographic findings can include focal wall thickening, intraluminal membranes, non-shadowing, and non-mobile intraluminal echogenic material [14,18]. CT scans may demonstrate contrast extravasation, high attenuation within the gallbladder lumen, and fluid-fluid layering [18].

The preoperative diagnosis of CCF is rare, accounting for only 7.9% of all CCF patients, compared to 43% of the series reporting cholecystoenteric fistulas in general [6,19,20]. Most CCFs are discovered intraoperatively as incidental findings during cholecystectomies. Furthermore, clinical diagnosis is difficult, as the signs and symptoms vary. Although abdominal pain in the right upper quadrant and diarrhea are most common, they can also present with nausea, vomiting, weight loss, and malabsorption [3,7,20,21].

Available abdominal radiography, abdominal ultrasonography, barium studies, biliary scintigraphy, and endoscopic retrograde cholangiopancreatography (ERCP) techniques have been used in cases of CCF [5,6,22]. In a recent case report, multidetector CT (MDCT) with intravenous contrast revealed a fistula with a diameter of 2 cm between the gallbladder and transverse colon [5]. Failure to identify CCF during surgery can lead to serious complications such as perforation of the colon, which can result in fecal peritonitis, sepsis, and even death in severe cases [6]. CCF can allow large stones to pass and cause bowel obstruction at the rectosigmoid diverticula, especially if the stone size is more than 2.5 cm [6,23].

For cholecystoenteric fistulas, surgical intervention is the treatment of choice; however, controversy exists between laparoscopic and open surgical approaches. Chowbey et al. reported that a large number of fistula cases approached laparoscopically had a conversion rate of 6.3% [19]. Moreover, many recent reports on CCFs have used laparoscopy and robotic approaches [24-26]. It is recommended that CCF should be resected if incidentally discovered during laparotomy, because there is a 15% risk of cholecystitis, cholangitis, and malignancy [6,22]. In our case, the decision to perform exploratory laparotomy was based on the patient’s condition and the findings of preoperative images. Cholecystectomy, right hemicolectomy, and ileocolic side-to-side anastomosis were performed successfully. The patient was discharged in a stable condition with no complications.

## Conclusions

Hemorrhagic cholecystitis and CCF require a high clinical suspicion. Early recognition and intervention are the cornerstones of management for avoiding life-threatening consequences. Careful review and consideration are required in patients presenting with right upper quadrant pain, positive Murphy’s sign, impaired liver function, and leukocytosis that use chronic anticoagulants or antiplatelet medications.
